# Pericapsular nerve group block versus fascia iliaca block for perioperative analgesia in hip fracture surgery: a prospective randomized trial

**DOI:** 10.11604/pamj.2023.46.93.41117

**Published:** 2023-11-29

**Authors:** Mariem Keskes, Mohamed Ali Mtibaa, Ameur Abid, Nizar Sahnoun, Salma Ketata, Rahma Derbel, Imen Zouche, Hichem Cheikhrouhou

**Affiliations:** 1 Department of Anesthesiology and Intensive Care Unit, Habib Bourguiba University Hospital, 3000 Sfax, Tunisia,; 2 Department of Orthopedic Surgery, Habib Bourguiba University Hospital, 3000 Sfax, Tunisia

**Keywords:** Pericapsular nerve group block (PENG), supra-inguinal fascia iliaca compartment block (SI-FICB), perioperative analgesia, hip fracture surgery

## Abstract

The aim of our study was to evaluate the efficacy of the pericapsular nerve group block (PENG) versus the supra-inguinal fascia iliaca compartment block (SI-FICB) to improve analgesia during positioning for spinal anesthesia (SA) for hip fracture surgery. We conducted a prospective randomized clinical trial involving patients who will undergo hip fracture surgery under SA and randomized into two groups: the PENG group: patients who received PENG block with 10 ml of 0.25% bupivacaine and 10 ml of 2% lidocaine and the SI-FICB group: patients who received SI-FICB block with the same solution. Our primary outcome was the Visual Analogue Scale (VAS) score at positioning for SA. Secondary outcomes were VAS after the block, the ease of spinal positioning (EOSP), the time to perform the block, the postoperative morphine consumption, and the VAS score at the 3^rd^, 6^th^, 12^th^, and 24^th^ postoperative hours. Eighty-nine patients were enrolled and randomized into two groups: 44 in the PENG group and 45 in the SI-FICB group. The time of block performance was comparable in both groups (p = 0.195). There was a significant decrease in pain scores in the 2 groups, 20 min after the blocks at rest and while positioning for SA. PENG block provided better analgesia than SI-FICB block at positioning (P=0.046) with no significant difference in the ease of positioning (p=0.328). The morphine consumption was comparable in the 2 groups (p = 0.842). There was no significant difference in VAS scores at the 3^rd^, 6^th^, 12^th^, and 24^th^ postoperative hours with p respectively 0.061, 0.767, 0.198, and 0.130. Both PENG and SI-FICB blocks provided adequate perioperative analgesia with the superiority of the PENG block in the sitting position for SA.

## Introduction

Hip fracture is a frequent orthopedic emergency, requiring surgery ideally within the first 72 hours [1]. It mostly affects elderly patients and is associated with significant morbidity and mortality [2,3]. Hip fractures cause significant pain in the preoperative and postoperative periods, especially when the hip is mobilized, which may exacerbate underlying cardiac, pulmonary, and cognitive diseases. No anesthetic technique has shown superiority to one over the other [4,5]. However, spinal anesthesia is preferred in orthopedic surgery due to its analgesic efficacy in the immediate post-operative period and its cost-effectiveness. The supra-inguinal fascia iliaca compartment block (SI-FICB) along with the femoral nerve block (FNB) proved their efficacy in providing immediate pain relief and adequate post-operative analgesia [6]. The pericaspular nerve group (PENG) block is a relatively new technique based on a recent anatomic study and has been proposed to provide analgesia in hip fracture patients. However, comparative studies between PENG and SI-FICB are lacking.

The aim of our study was to compare the efficacy of the pericapsular nerve group block (PENG) versus the supra-inguinal fascia iliaca compartment block (SI-FICB) to improve analgesia during positioning in spinal anesthesia.

## Methods

This is a clinical trial being conducted in the anesthesia-intensive care unit, which follows a prospective controlled, simple-blinded, and randomized design. The study focused on patients who were scheduled for surgery on the upper extremity of the femur under spinal anesthesia (SA).

**Study population:** we included patients aged over 65 years undergoing hip fracture surgical repair under spinal anesthesia with American Society of Anesthesiologists (ASA) score of I, II, or III, and a preoperative visual analogue scale (VAS) score over 5 during mobilization of the fractured limb. Patients who met any of the following criteria were not included in the study: contraindications to spinal anesthesia, patient refusal, known allergy to local anesthetics, and misunderstanding of the visual analog scale. Additionally, patients were excluded from the study if they experienced a failure of spinal anesthesia, necessitating a conversion to general anesthesia (GA), or if they did not adhere to the study protocol.

**Sample size:** since there was no clinical trial evaluating PENG block in the reduction of the VAS score at positioning for spinal anesthesia, the sample size for this study was determined based on a preliminary survey involving 20 patients. A decrease of 15% in the visual analog scale (VAS) score during the positioning for spinal anesthesia (SA) was considered clinically significant. The calculation was performed using a significance level (alpha) of 5% and a study power of 90%, which resulted in a minimum required sample size of 30 patients in each group. To account for potential exclusions during the study, a total of 45 patients per group were designated to ensure a sufficient number of participants.

**Randomization and allocation:** in this study, patients were randomized into two groups using a sequence generated by the sealedenvelope website. The randomization process aimed to ensure an unbiased distribution of participants among the groups, facilitating a fair comparison of the outcomes. The two groups were: PENG group: patients who received pericapsular nerve group block with 10 ml of 0.25% bupivacaine and 10 ml of 2% lidocaine and SI-FICB group: patients who received supra-inguinal fascia iliaca compartment block (SI-FICB) with 10 ml of 0.25% bupivacaine and 10 ml of 2% lidocaine.

**Interventions:** a checklist was performed in the operating room and the patient´s identity and side to operate on are verified. Patients were then monitored by electrocardioscope, pulse oximetry, and non-invasive blood pressure monitoring. An 18-gauge vein needle was placed and patients received 10 cc/kg of 0.9% of ringer lactate. Pre-procedure pain was assessed during rest (T1) as well as on movement (T2) on 15° passive elevation of the affected limb and recorded via visual analog pain scale (VAS) (0 = no pain; 10 = worst imaginable pain) previously explained to the patient. Patients were then randomized to either receive PENG block or SI-FICB. The solution of the local anesthetic compromised 10 ml of 0.5% bupivacaine et 10 ml of 2% lidocaine. The blocks were performed by an experienced anesthesiologist in the supine position and a strict sterile technique was followed. The PENG block was performed using a linear probe of 8 MHZ, after identification of the psoas muscle and its hyperechogenic tendon, the anterior inferior iliac spine, and ilio-pubic eminence. A needle 25 gauge 100 mm was inserted in the plane between the psoas muscle tendon and the ilio-pubic eminence and the solution was injected in increments of 5 ml until we see the elevation of the psoas muscle. The SI-FICB was performed using a linear probe of 8 MHZ that was placed over the inguinal ligament, close to the anterior superior iliac spine, and oriented in the para-sagittal plane. The probe is then moved supero-laterally along the inguinal ligament until the anterio-iliac spine is imaged. The deep circumflex iliac artery is identified as superficial to the fascia iliaca. The needle is then introduced in plane parallel to the probe and a ‘pop´ is usually felts when it passes through the fascia iliaca.

The position is confirmed by an injection of 1 ml of local anesthetic and a lens deep to the fascia is formed. The rest of the solution is injected in increments of 5 ml. The time to perform the block, which is the time required to image the landmarks and inject the solution, was noted. Twenty minutes after the blocks, analgesia was measured by VAS at rest (T3) and while positioning the patient for SA (T4). The ease of spinal positioning (EOSP) was graded as optimal (the patient easily assumed a sitting position without any assistance), easy (the patient struggled to assume a sitting position without any assistance), and difficult (the patient had difficulty assuming a sitting position and required assistance). The spinal anesthesia procedure was performed with the patient in the sitting position, targeting the L4-L5 or L5-S1 intervertebral space using a midline approach and a 25-gauge Quincke spinal needle. Each patient received 10 mg of 0.5% hyperbaric bupivacaine along with 5 μg of sufentanil for the anesthesia.

After the successful administration of spinal anesthesia, patients were then positioned in a supine position. To ensure proper oxygenation, oxygen was administered at a rate of 3 liters per minute through a face mask. This step aimed to maintain adequate oxygen levels during the anesthesia procedure and subsequent post-anesthesia period. Surgery was authorized when an anesthesia metameric level of T6 was reached, otherwise, we converted to general anesthesia, and the patient was then excluded from the study.

After the surgery, the patient was transferred to the Post Interventional Monitoring Room (PIMR) for 2 hours where postoperative analgesia was provided by: 1 g of paracetamol and 2 mg morphine titration every 10 minutes if the VAS value was greater than 3. After that, patients were transferred to their referral department. Postoperative analgesia was provided with IV paracetamol 1 gm every 8 h. The rescue analgesia was provided with 50 mg tramadol when VAS was >3. VAS score was evaluated at the 3^rd^, 6^th^, 12^th^, and 24^th^ postoperative hours.

**Data collection:** pre- and intraoperative data were collected including patients´ demographics, ASA score, and duration of surgery. VAS before the block at rest (VAS T1) and at mobilization (VAS T2), at 20 minutes after block at rest (VAS T3) and in the sitting position (VAS T4), ease of patient positioning (EOSP) as well as time to perform the block were also recorded. We collected post-operative anesthetic and analgesic parameters such as VAS at the 3^rd^, 6^th^, 12^th^, and 24^th^ postoperative hours, the average dose of morphine consumption in the PIMR, and the presence of complications. The collection of intra-operative and post-operative data was done by an anesthesiologist different than who performed the block.

**Variables:** the primary outcome was the VAS score at positioning for SA. Secondary outcomes were VAS score at the rest 20 minutes after the block, the EOSP, time to perform the block, morphine consumption in the PIMR, VAS scores at the 3^rd^, 6^th^, 12^th^, and 24^th^ postoperative hours, and the presence of side effects.

**Statistical analysis:** for data analysis, we utilized SPSS version 25.0 software. The normality of the distribution for quantitative variables was assessed using the Shapiro-Wilk test. Continuous variables with a normal distribution were presented as means with standard deviation (SD), while those without a normal distribution were expressed as medians with semi-interquartile ranges (SIR). Qualitative variables were presented as frequency distributions. To compare the two groups of patients, univariate analyses were performed using various statistical tests. The t-student test was used for continuous variables with a normal distribution, the Mann-Whitney test for continuous variables without a normal distribution, and Pearson's Chi-square test for qualitative variables. Statistical significance was considered to be achieved when the p-value was less than 0.05 (p < 0.05).

**Ethical considerations:** this study was conducted with the approval of the Southern Protection Committee of People (C.P.P.SUD) under the supervision of the Health Ministry of the Tunisian Republic. The reference for this approval was CPP SUD N°0506/2023, which was obtained based on the nature of the product and the study's methodology.

Furthermore, the study was carried out with the explicit written informed consent of all participating patients. Prior to their involvement in the research, patients were provided with comprehensive information about the study's objectives, procedures, potential risks, and benefits. They voluntarily agreed to participate after understanding the details and implications, ensuring their full informed consent throughout the duration of the study.

## Results

**Figure 1:**
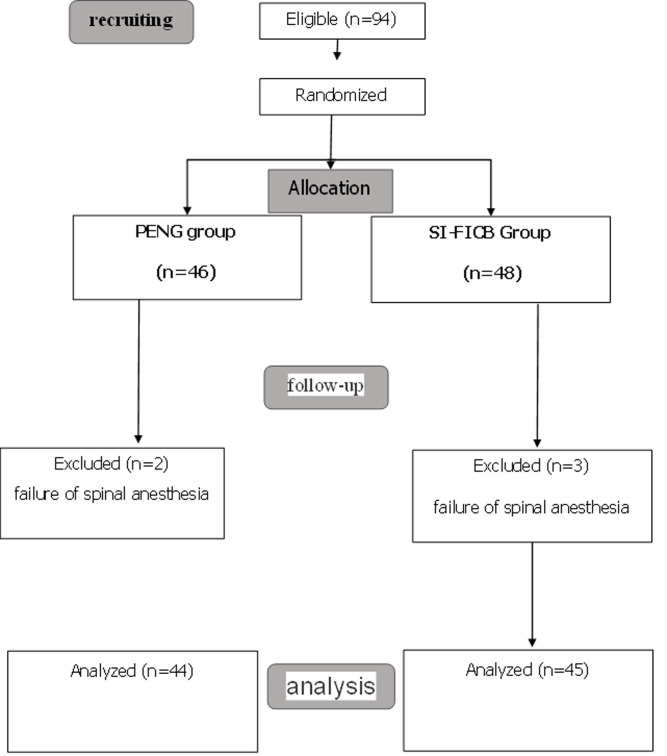
flowchart depicting the study participants recruited from the cardiology consultation at the teaching hospital in Bandas City, Senegal, spanning from June 2019 to December 2020 (N=450)

Ninety-four patients were included in our study and were divided into two groups. Two patients from the SI-FICB group and three patients from the PENG group were excluded due to spinal anesthesia (SA) failure. The final sample size consisted of eighty-nine subjects, with forty-four in the PENG group and forty-five in the SI-FICB group (Figure 1).

**General characteristics:** the mean age of the study population was 76.51 ± 8.8 years old. The sample consisted of 41 females and 48 males with a sex ratio of 1.17. The mean body mass index (BMI) was 25.9 ± 3.5 kg/m^2^. The demographic variables were comparable between the two groups (Table 1).

**Table 1:** comparison of demographic data between the two groups

Demographic parameters	SI-FICB group (N=45)	PENG group (N=44)	P value
Age (years) ±SD	77.7 ± 8.9	75.3 ± 8.6	0.170*
Weight (kg) ±SD	71.6 ± 7.2	75 ± 9.7	0.093*
Height (cm) ±SD	167.91 ± 5.6	169.18 ± 7.75	0.132*
BMI (kg/m2) ±SD	25.48 ± 3.01	26.34 ± 3.98	0,250*
Sex (M/F)	20/25	28/16	0,069ƚ
ASA score (I/II/III)	5/38/2	7/31/6	0,220ƚ

SD: standard deviation; PENG: pericapsular nerve group block; SI-FICB: supra-inguinal fascia iliaca compartment block; cm: centimeter; Kg: kilogram; N: number; M: male; F: female; *: t-student test, ƚ: Pearson’s Chi-square test; ASA: American Society of Anesthesiologists

**Intraoperative analgesia:** the pre-block VAS scores in both groups were comparable at rest and on movement (P = 0.177, P = 0.065, respectively) (Table 2).

**Table 2:** comparison of VAS scores at pre-block and 20 minutes post-block between the two groups

Parameters	SI-FICB group(N=45)	PENG group(N=44)	P value
VAS T1 ± SD	5.84 ± 0.82	6.11 ± 0.92	0.177*
VAS T2 ± SD	8.24 ± 0.98	8.64 ± 0.83	0.065*
VAS T3 ± SD	0.84 ± 0.706	0.61 ± 0.493	0.078*
VAS T4 ± SD	2.16 ± 0.824	1.82 ± 0.582	0.046*
P1 value	p < 0.01	p < 0.01	
P2 value	p < 0.01	p < 0.01	

SD: standard deviation; PENG: pericapsular nerve group block; SI-FICB: supra-inguinal fascia iliaca compartment block; N: number; VAS T1: VAS before the block at rest; VAS T2: VAS before the block at mobilization; VAS T3: VAS at 20 minutes after block at rest; VAS T4: VAS at 20 minutes after block in sitting position; *: test student; P value: SI-FICB group versus PENG group; P1 value: VAS T1 versus VAS T3; P2 value: VAS T2 versus VAS T4; VAS: visual analogue scale

Twenty minutes after block, in the PENG group, the VAS score at rest decreased significantly from 6.11 ± 0.92 to 0.61 ± 0.7 (p < 0.01) and at mobilization from 8.64 ± 0.83 to 1.82 ± 0.582 (p < 0.01). In the SI-FICB group, the VAS score at rest decreased significantly from 5.84 ± 0.82 to 0.84 ± 0.7 (p < 0.01), and at mobilization from 8.24 ± 0.98 to 2.16 ± 0.824 (p < 0.01) (Table 2). There was no significant difference between the two groups post-block at rest (p=0.078). The VAS score while positioning for the spinal anesthesia was significantly lower in the PENG group (1.82 ± 0.582) versus the SI-FICB group (2.16 ± 0.824) with p = 0.046 (Table 2).

Time of block performance was comparable in both groups, 4.32 ± 0.98 min for the PENG group and 4.2 ± 1.42 min for the SI-FICB group (p = 0.195). The EOSP was comparable between the two groups, 35 patients of the PENG group (79.5%) and 33 patients of the SI-FICB group (73.3%) had easy to optimal positioning (p=0.328) (Table 3).

**Table 3:** comparison of EOSP scores between the two groups

Parameters	SI-FICB group(N=45)	PENG group(N=44)	P value
Optimal	9 (20%)	5 (11.3%)	0.328ƚ
Easy	24 (53.3%)	30 (68.1%)	
Difficult	12 (26.6%)	9 (20.45%)	

N: number; SI-FICB group: supra-inguinal fascia iliaca compartment block group; PENG group: pericapsular nerve group block group; EOSP: ease of spinal positioning; ƚ: Pearson’s Chi-square test

**Postoperative analgesia:** the mean morphine consumption in the PIMR for patients in the SI-FICB group was 1.33 mg ± 2.17, compared to 1.18 mg ± 2 in the PENG group, with no statistically significant difference (p = 0.842).

There was no significant difference in VAS scores at the 3^rd^, 6^th^, 12^th^, and 24^th^ postoperative hours between the two groups with p respectively 0.061, 0.767, 0.198, and 0.130 (Table 4). None of the patients reported any block-related complications.

**Table 4:** comparison of postoperative VAS scores between the two groups

Parameters	SI-FICB group (N=45)	PENG group (N=44)	P value
VAS (H3) ± SD	1.67 ± 0.78	2.02 ± 0.92	0.064*
VAS (H6) ± SD	2.47 ± 0.78	2.7 ± 1.39	0.767*
VAS (H12) ± SD	3.33 ± 1	3.07 ± 1.14	0.198*
VAS (H24) ± SD	3.02 ± 0.91	3.45 ± 1.35	0.130*

SD: standard deviation; SI-FICB: supra-inguinal fascia iliaca compartment block group; PENG: pericapsular nerve group block group; N: number; *: student test; VAS: visual analogue scale

## Discussion

We conducted a prospective simple-blinded randomized clinical trial including patients proposed for upper femur surgery under spinal anesthesia to evaluate the efficacy of the pericapsular nerve group block (PENG) versus the supra-inguinal fascia iliaca compartment block (SI-FICB) to improve perioperative analgesia. Eighty-nine patients were analyzed and randomized into two groups: forty-four in the PENG group and forty-five in the SI-FICB group. The time of block performance was comparable in both groups. This study showed that both PENG and SI-FICB block provided adequate perioperative analgesia. We noted a significant decrease in pain scores in the 2 groups, 20 min after the blocks at rest and while positioning for SA when comparing them with the pain scores before blocks. PENG block provided better analgesia than SI-FICB block at positioning for SA with no significant difference in the ease of positioning.

Spinal anesthesia (SA) is the preferred choice for performing surgery in cases of hip fractures, particularly in elderly patients with additional medical co-morbidities. However, achieving the optimal sitting position for SA can be challenging due to the severe pain experienced by these patients, which can potentially exacerbate their co-morbidities. PENG block is a relatively new regional analgesic technique first described in 2018 by Girón-Arango *et al*. [7]. PENG block targets the articular sensory branches to the anterior hip joint with a single injection based on the cadaveric study that showed a significant contribution of the accessory obturator nerve (in addition to femoral and obturator nerves) towards anterior hip joint innervations [8]. Following the initial description of the PENG block, there have been a significant number of publications of case reports and case series highlighting the excellent analgesic benefit of this block for perioperative analgesia in hip surgery [9-15]. Few studies examined the role of its analgesic efficacy in the preoperative setting. Girón-Arango *et al*. [7] published a case series study that included 5 patients with hip fractures who received PENG block and proved a significant pain reduction at rest and movement 30 minutes post-block. Another case series published by Achraya *et al*. [14] including 10 patients who received PENG block for hip fracture proved significant pain relief at rest and movement 10 minutes post-block.

Supra inguinal fascia iliaca block is a relatively new approach first described by Hebbard *et al*. in 2011 [16]. This technique utilizes the anterior superior iliac spine as a reference point to locate the fascia iliaca and iliac muscle [17,18]. In this method, the needle is directed cephalad, facilitating the upward diffusion of the analgesic and ensuring a more comprehensive blockage of the obturator and lateral femoral cutaneous nerves. A clinical trial conducted on elderly patients (over 65 years old) undergoing hip replacement surgery reported that supra-inguinal FICB effectively provided pain relief and potentially reduced the need for opioid medication compared to the traditional FICB [19]. Additionally, another study demonstrated that the supra-inguinal approach resulted in a more pronounced blockage of the lateral femoral cutaneous nerve when compared to the conventional approach [20].

Our study showed that the PENG block provided better analgesia than the SI-FICB block at positioning for SA. One prospective randomized study published by Jadon *et al*. [21] which included 66 patients comparing the PENG block and SI-FICB, proved the superiority of the PENG block in providing better analgesia when positioning for SA.

We compared the performance time of the two blocks and did not find a significant difference between the two groups. Aliste *et al*. [22] demonstrated the absence of a significant difference between the performance time of the two blocks: 4.4 minutes (1.8) for PENG vs 5 minutes (1.6) for BIFSI, with a p-value of 0.230.

In our study, the postoperative VAS scores were compared at H3, H6, H12, and H24, and the results did not show a statistically significant difference between the two groups. Aliste *et al*. [22] compared the postoperative VAS and did not find a statistically significant difference between the two groups at H3, H6, H12, and H24 postoperatively. However, a study published by Jadon *et al*. [21], with the secondary objective of VAS scores at H4, H6, H12, and H24 at rest and during mobilization, showed an advantage for the SI-FICB group at H12 at rest and H24 during mobilization.

**Limitations:** our study had several main limitations that need to be acknowledged. First, there was inter-individual variability in pain perception among the patients, which could have influenced the results. Pain perception is subjective and can vary significantly from person to person, potentially affecting the interpretation of the findings. Secondly, the evaluation of the visual analog scale (VAS) score relied on subjective reporting by the patients themselves. The accuracy of the VAS score could be influenced by factors such as the intellectual level of the patients, especially if they were elderly subjects. This might have introduced some degree of variability in the pain assessment. Lastly, due to practical constraints, it was not possible to have the same surgeon operate on all patients. Different surgeons may have slightly different techniques or approaches, which could have introduced variability in the surgical outcomes. It is important to consider these limitations when interpreting the results of our study, as they could have impacted the overall conclusions and generalizability of the findings.

## Conclusion

Both PENG and SI-FICB blocks provided adequate perioperative analgesia with the superiority of the PENG block in the sitting position for spinal anesthesia.

### What is known about this topic

- *Hip fractures cause significant pain in the preoperative and postoperative periods;*
- *Spinal anesthesia is preferred in hip fractures surgery due to its analgesic efficacy in the immediate post-operative period;*
- *The supra-inguinal fascia iliaca compartment block provides immediate pain relief and adequate postoperative analgesia; the pericaspular nerve group block is a relatively new technique that has been proposed to provide analgesia in hip fracture patients*.

### What this study adds

- *Both PENG and SI-FICB block provided a significant decrease in intraoperative pain scores;*
- *PENG block provided better analgesia than SI-FICB block at positioning for SA with no significant difference in the ease of positioning;*
- *The postoperative analgesic effect of PENG and SI-FICB block is comparable*.
